# Personalized management of differentiated thyroid cancer in real life – practical guidance from a multidisciplinary panel of experts

**DOI:** 10.1007/s12020-020-02418-x

**Published:** 2020-08-09

**Authors:** Alfredo Campennì, Daniele Barbaro, Marco Guzzo, Francesca Capoccetti, Luca Giovanella

**Affiliations:** 1 Department of Biomedical and Dental Sciences and Morpho-Functional Imaging, Nuclear Medicine Unit, University of Messina, Messina, Italy; 2 U.O. Endocrinologia, ASL Nord Ovest Toscana, Livorno, Italy; 3 Head and Neck Surgery Department, Fondazione IRCCS Istituto Nazionale dei Tumori, Milan, Italy; 4 Service Department Macerata Hospital, ASUR Marche AV3, Nuclear Medicine Unit, Macerata, Italy; 5 Clinic for Nuclear Medicine and Competence Centre for Thyroid Diseases, Ente Ospedaliero Cantonale, Bellinzona, Switzerland; 6 Clinic for Nuclear Medicine, University Hospital and University of Zurich, Zurich, Switzerland

**Keywords:** Differentiated thyroid carcinoma, Martinique Principles, Radioiodine therapy, Recommendations, Surgery

## Abstract

**Purpose:**

The standard of care for differentiated thyroid carcinoma (DTC) includes surgery, risk-adapted postoperative radioiodine therapy (RaIT), individualized thyroid hormone therapy, and follow-up for detection of patients with persistent or recurrent disease. In 2019, the nine Martinique Principles for managing thyroid cancer were developed by the American Thyroid Association, European Association of Nuclear Medicine, Society of Nuclear Medicine and Molecular Imaging, and European Thyroid Association. In this review, we present our clinical practice recommendations with regard to implementing these principles in the diagnosis, treatment, and long-term follow-up of patients with DTC.

**Methods:**

A multidisciplinary panel of five thyroid cancer experts addressed the implementation of the Martinique Principles in routine clinical practice based on clinical experience and evidence from the literature.

**Results:**

We provide a suggested approach for the assessment and diagnosis of DTC in routine clinical practice, including the use of neck ultrasound, measurement of serum thyroid-stimulating hormone and calcitonin, fine-needle aspiration, cytology, and molecular imaging. Recommendations for the use of surgery (lobectomy vs. total thyroidectomy) and postoperative RaIT are also provided. Long-term follow-up with neck ultrasound and measurement of serum anti-thyroglobulin antibody and basal/stimulated thyroglobulin is standard, with ^123^/^131^I radioiodine diagnostic whole-body scans and ^18^F-fluoro-2-deoxyglucose positron emission tomography/computed tomography suggested in selected patients. Management of metastatic DTC should involve a multidisciplinary team.

**Conclusions:**

In routine clinical practice, the Martinique Principles should be implemented in order to optimize clinical management/outcomes of patients with DTC.

## Introduction

**Fig. 1:**
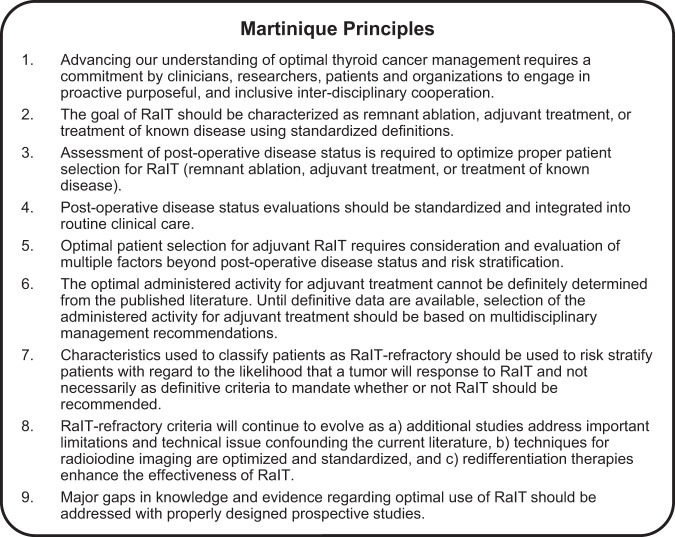
The Martinique principles summarizing the major points of discussion during the first Martinique meeting. Reproduced with permission from R.M. Tuttle et al. Thyroid (2019) [6]. RaIT radioiodine therapy

Differentiated thyroid cancer (DTC), most commonly the papillary histotype, accounts for the majority of thyroid cancer cases [1]. The standard of care for DTC includes surgery, risk-adapted postoperative radioiodine therapy (RaIT), and individualized thyroid hormone therapy tailored to the patient’s risk of relapse [2]. These approaches lead to excellent responses in >80% of patients [2]. However, as DTC carries a significant risk of disease persistence and recurrence, long-term active follow-up is essential [3, 4]. In 2015, the American Thyroid Association (ATA) recommended less intense treatment strategies for many DTC patients, including observation or thyroid lobectomy without RaIT [3], which resulted in controversy and significant differences in clinical practice [5]. In 2019, a joint statement from the ATA, European Association of Nuclear Medicine (EANM), Society of Nuclear Medicine and Molecular Imaging (SNMMI), and European Thyroid Association (ETA) defined the indications and practical issues of RaIT and agreed on a set of nine principles (the so-called “Martinique Principles”; Fig. 1) [6]. A multidisciplinary panel of five thyroid cancer experts debated the implementation of the Martinique Principles in routine clinical practice, with discussion undertaken via conference call and electronic communication. This paper presents our shared practical recommendations regarding DTC diagnosis, treatment with surgery and RaIT, and long-term follow-up.

## Differentiated thyroid cancer diagnosis

Thyroid nodules are frequently detected in the general population and usually have a low risk of malignancy. When a thyroid nodule is detected (either clinically or incidentally), medical history and physical examination, measurement of thyroid-stimulating hormone (TSH) levels and neck ultrasonography are advised. In addition, measurement of serum calcitonin should be considered to further delineate the possibility of medullary thyroid carcinoma, despite there being a lack of general consensus with regard to routine serum calcitonin measurement [3]. In the presence of reduced or low-normal TSH levels, thyroid scintigraphy is recommended as malignancy is very unlikely in autonomously functioning (“hot”) nodules [7]. Notably, although autonomous nodules are almost invariably accompanied by decreased TSH levels (i.e., <0.1–0.4 mUI/L) when iodine supply is adequate, the bulk of autonomous tissue may be insufficient to suppress the TSH level in iodine-depleted thyroids, especially in the early phases of autonomy [8]. As a consequence, the decision on whether or not to use thyroid scans should take into account the patient’s geographic location and the iodine supply in that area. Independently from the patient’s iodine status and the TSH levels, a thyroid scan is also recommended in people with a large multinodular goiter to select suspicious nodules for fine-needle aspiration (FNA), and in people with cytologically indeterminate nodules (i.e., follicular proliferation) to identify a benign compensated functioning adenoma [9].

In nonautonomous nodules, FNA is currently recommended based on ultrasound characteristics. Considerable effort has been made toward the standardization of both ultrasound and FNA cytology reports, with the development of different ultrasound and cytological reporting systems [i.e., TIRADS for ultrasound and the Italian consensus for the classification and reporting of thyroid cytology (SIAPEC)] [10, 11]. Despite such efforts, up to 25% of nodules are still considered to be “cytologically indeterminate”, requiring in many cases diagnostic surgery to definitely confirm or exclude malignancy (with a prevalence for malignant nodules of about 30%). Immunocytochemistry, molecular testing (i.e., mutation analysis, microRNA) [12, 13], and imaging techniques, including ^99m^Tc-methoxy-isobutyl-isonitrile (MIBI) scintigraphy and ^18^F-fluoro-2-deoxyglucose positron emission tomography/computed tomography (^18^F-FDG-PET/CT) [14–17] may be useful in these cases to avoid unnecessary surgeries. Indeed, none of such methods had both near-perfect sensitivity and specificity. Notwithstanding, test performance strongly depends on population-dependent variations in cytology, tumor genetics, and the prevalence of malignancy as well as on the costs and feasibility of the desired diagnostic protocol in the local patient population. Accordingly, the choice of one or more additional test should be a deliberate and multidisciplinary one [18].

**Fig. 2:**
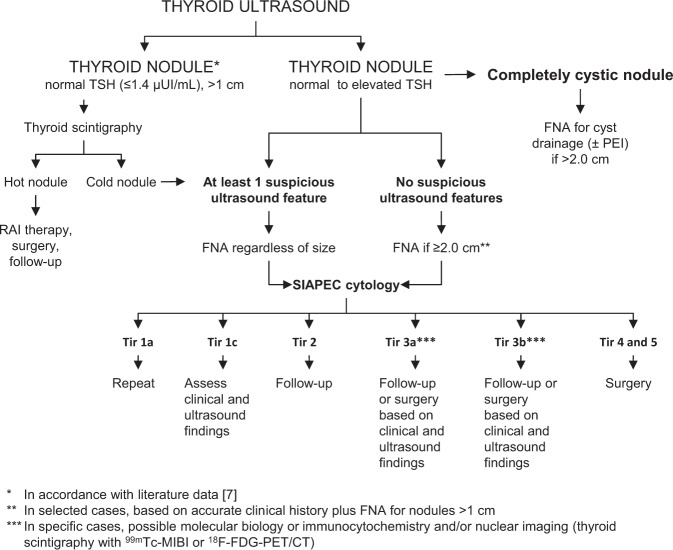
Diagnostic algorithm for patients presenting with thyroid nodules. ^18^F-FDG-PET/CT ^18^F-fluoro-2-deoxyglucose positron emission tomography/computed tomography, DTC differentiated thyroid carcinoma, FNA fine-needle aspiration, MIBI methoxy-isobutyl-isonitrile, PEI percutaneous ethanol injection, RAI radioiodine, SIAPEC Italian Societies of Endocrinology and Pathology, TSH thyroid-stimulating hormone

Figure 2 summarizes our suggested approach to assessing thyroid nodules in routine clinical practice.

### Key points and practical indications

- Primary diagnostic assessments include neck ultrasound, serum TSH, FNA cytology, and (optional) calcitonin measurement.
- Thyroid scintigraphy (with ^99m^Tc pertechnetate or ^123^I) is recommended when TSH levels are reduced (<0.3–0.4 mIU/L) and can be considered for TSH levels up to 1.0–1.5 mIU/L depending on local iodine supply.
- FNA should not be performed in autonomously functioning thyroid nodules confirmed by thyroid scintigraphy, with possible exceptions being clinically or ultrasonographically highly suspicious nodules.
- Using a standardized system to report thyroid ultrasound findings is recommended to provide consistent communication; however, the ultrasonographers’ expertize remains pivotal to translate ultrasound results into clinical actions.
- Based on our experience, FNA is suggested when any one of these ultrasound features is present: hypoechoic nodule; nodule with blurred/irregular margins; nodules with microcalcifications (<2 mm); or nodule that is taller than it is wide.
- In the absence of clearly suspicious ultrasound features, FNA cytology should be considered based on diameter (i.e., >1–2 cm), clinical history, and physical examination.
- Molecular testing and molecular imaging based on ^99m^Tc-MIBI scintigraphy and ^18^F-FDG-PET/CT may help in refining the diagnosis in selected cases of nodules with indeterminate cytology.

## Thyroid surgery

According to the 2015 ATA guidelines, lobectomy may be sufficient for a unifocal intrathyroidal low-risk carcinoma of <4 cm diameter in patients with no prior head and neck radiation, familial thyroid carcinoma, or clinically detectable cervical lymph node metastases, whereas in other cases, total or near total thyroidectomy is recommended [3]. Approximately 20–50% of patients with DTC have cervical lymph node metastases, mostly in the central neck (i.e., VI Robbins’ level) [19]. Central compartment dissection is a well-established treatment of clinical N1a (cN1a) and, coupled with lateral dissection, N1b (cN1b) disease, although its role as prophylaxis in cN0 disease is unclear [3]. Additional factors, including lesion location, multifocality, bilaterality, thyroid capsule invasion, and *BRAF*^V600E^ and *TERT* mutation status, should also be considered to refine surgical planning [20–22].

Overall, we agree with the ATA 2015 recommendations on neck dissection and total thyroidectomy in intermediate- to high-risk DTC; however, the use of lobectomy for low- to intermediate-primary tumors ≤4 cm may be too broad. Current controversies on the extent of surgery probably arise from heterogeneous studies [23]. Notably, the risk for local and distant metastases increases with increasing size of the papillary thyroid cancer (PTC), especially if the tumor size exceeds 2 cm, as has been described in an observational study involving 366 patients with PTC [24].

Moreover, data from the United States National Cancer Data Base showed that patients with papillary DTC sized 2.0–3.9 cm had improved survival when treated with total thyroidectomy [25]. Accordingly, the Swiss consensus on low-risk papillary DTC limited the definition of low-risk DTC, treatable with lobectomy, to cancer with low-risk ATA 2015 characteristics but with tumor size <2 cm [26].

### Key points and practical indications

- More evidence is needed before generalizing the current trend towards use of lobectomy for low-risk primary tumors ≤4 cm in clinical practice. Thus, it may be safer to restrict lobectomy to low-risk tumors <2 cm.
- In other cases (i.e., low-risk >2 cm, intermediate- and high-risk DTC), we recommend (near) total thyroidectomy as the first-line procedure, followed by RaIT (discussed below).
- Central compartment dissection is a well-established treatment of cN1a tumors; however, because its role as prophylaxis in cN0 tumors is uncertain we recommend its use should be avoided in this setting (especially in T1-2 tumors).
- Lateral neck dissection is only indicated in cN1b tumors (coupled with central neck dissection).

## Postoperative radioiodine therapy

Following surgery, the risk of structural disease recurrence and/or persistence should be assessed using the three-tier (low, intermediate, and high) stratification suggested by the ATA in 2009 [27] and modified in 2015 [3], while the risk of mortality from thyroid cancer is estimated using the AJCC/TNM staging system [28]. RaIT has long been the standard of care following primary surgery for all patients with DTC, except those with unifocal PTC < 1 cm and no high-risk characteristics [4]. Based on 2015 ATA guidelines, routine RaIT is recommended for high-risk patients, suggested for intermediate-risk patients, and restricted to selected low-risk patients [3]. However, Tuttle et al. recognized that the actual goal of RaIT (i.e., ablation of thyroid remnant, adjuvant therapy, and treatment of known disease) can only be determined once the postoperative disease status has been assessed [6]. Then, a comprehensive assessment of risk and postoperative disease status is needed to decide whether RaIT is necessary or if observation will be sufficient. Patients with evidence of persistent disease after appropriate initial surgery may be the only candidates for ‘treatment of known disease’, regardless of initial risk stratification. In other cases, patients may be candidates for observation, remnant ablation, or adjuvant treatment based on careful risk assessment, as well as patients’ preferences. However, there are currently no reliable, universally accepted, precise recommendations to guide the proper assessment of postoperative disease status. Until precise, evidence-based guidelines are available, multidisciplinary teams should establish local standards to guide clinical management that considers the availability and quality of pre- and post-operative imaging and thyroglobulin (Tg) measurements, the experience of operating surgeon, and local clinical concerns [3]. In this setting, it should be considered that whole body scintigraphy (WBS) with single-photon emission computed tomography/CT (SPECT/CT) obtained after administration of therapeutic ^131^I-radioiodine (RAI) activity (i.e., >1.1 GBq) and preablation stimulated Tg measurement remain the most accurate tools for postoperative DTC restaging and are also included in ATA risk stratification system [3].

Accordingly, our panel members agreed on recommending adjuvant RaIT for all patients with high- and intermediate-risk cancer, since it allows for early diagnosis of residual disease and reduces the risk of recurrence [29–32]. In addition, as the risk of local and distant metastases increases with increasing size of the PTC, especially in tumors >2 cm, the same approach is suggested in low-risk DTC patients with a primary tumor >2 cm [26]. Importantly, patients who receive RaIT for ablation or adjuvant purposes with no signs of persistent disease (i.e., undetectable Tg, negative ultrasound and, if performed, negative diagnostic WBS) could be fully reassured and simply monitored every 1–2 years by clinical examination and basal Tg measurement avoiding Tg stimulation tests or periodic neck ultrasound examination during follow-up. Finally, adjuvant treatment is not routinely recommended for low-risk patients with a primary tumor <2 cm; however, if additional risk factors (i.e., isthmic location, known *BRAF* mutation) or increased Tg antibody (TgAb) levels are present or a preference for maximum-intensity treatment is expressed by patients, RaIT for remnant ablation is an option.

### Key points and practical indications

- RaIT has three distinct goals: remnant ablation, adjuvant treatment, or treatment of known residual or recurrent disease.
- For low-risk DTC patients with a primary tumor size <2 cm and neither additional risk factors nor elevated TgAb, we recommend observation; routine RaIT is not indicated. Patients who request maximum-intensity treatment, after being informed about their low but existing risk for recurrence, can be treated with RaIT for remnant ablation.
- For patients with low-risk DTC and a primary tumor size of 2–4 cm or additional risk factors, and for patients with intermediate- or high-risk DTC, routine adjuvant RaIT is recommended.
- Pretreatment stimulated Tg and posttreatment Dx-WBS-SPECT/CT should always be obtained and serve as platform for restaging and follow-up planning.

### Postoperative RaIT for remnant ablation or adjuvant treatment

We generally recommend reducing the daily intake of iodine-containing food for 2 weeks before initiating RaIT [33–36]. Dietary recommendations are listed in Table 1. This is particularly relevant for patients with euthyroid status stimulated with recombinant human TSH (rhTSH) [37]. Measurement of urine iodine content may also be useful, as an association between high urinary iodine excretion and the failure of RaIT has been reported [38].

**Table 1:** Recommendations for reducing iodine intake

*SUMMARY*
• No iodized salt
• No dairy products or foods containing dairy products
• No foods from the sea
• Limit grain products (i.e., noodles, pasta, and pastries) to 1 slice of bread, ½ cup of pasta daily
• Limit the amount of beef, chicken, and turkey
*FOODS TO AVOID*
• Iodized salt
• Any vitamins or supplements containing iodine (especially kelp and dulse)
• Milk or other dairy products, including ice cream, cheese, yogurt, and butter
• Seafood, including fish, sushi, shellfish, kelp, and seaweed
• Herbal supplements
• Foods containing the additives carrageen, agar-agar, alginate, or nori
• Commercially prepared bakery products made with iodate dough conditioners
• FD & C red dye #3, which is found in maraschino cherries and occasionally as a pink/red artificial color in beverages
• Egg yolks, whole eggs, and foods containing whole eggs
• Milk chocolate (due to dairy content)
• Blackstrap molasses (unsulfured molasses is fine)
• Soy products (soy sauce, soy milk, tofu) as high soy ingestion has been shown to interfere with RAI uptake
*FOODS TO EAT*
• Non-iodized salt or non-iodized sea salt
• Egg whites
• Homemade bread made with non-iodized salt and oil (not soybean oil, butter, or milk) or commercially baked breads that do not contain iodate dough conditioners, dairy, or eggs
• Fresh fruits and vegetables
• Frozen vegetables
• Grain, cereal products, and pasta without high iodine ingredients
• Canned fruit
• Natural unsalted nuts and nut butters (e.g., peanut or almond)
• Sodas, beer, wine, lemonade, and fruit juices
• Coffee or tea (without milk, cream, or soy-based nondairy creamer)
• Popcorn popped in vegetable oil or air popped with non-iodized salt
• Black pepper, fresh or dried herbs and spices, and all vegetable oils
• Sugar, jam, jelly, honey, and maple syrup
• Matzoh crackers
*POSSIBLE SOURCES OF IODINE INTERFERENCE*
• Iodine-containing multivitamins (for 7 days after use)
• Iodine-containing disinfectants, toothpaste, or vaginal lavages (for 2–3 weeks)
• Iodine tincture (for 2–3 weeks)
• Water-soluble iodinated contrast agent (for 3 months) or oil-soluble iodinated contrast agent (for ≥3 months)
• Amiodarone (for 6 months or more if obese)
• Valpressin (for several weeks; suggest testing for ioduria before RaIT)
*ADDITIONAL GUIDELINES*
• Avoid restaurant foods since there is no reasonable way to determine whether they use iodized or non-iodized salt.
• Consult your doctor before discontinuing any red-colored medication or any iodine-containing medication (e.g., amiodarone, expectorants, or topical antiseptics).
• Avoid all herbal supplements (especially when it is unclear how much iodine they contain).

*RAI* radioiodine, *RaIT* radioiodine therapy

Conflicting data area reported on RAI activities that should be used for ablative purpose [39–42]. In general, RAI activities of around 1.1–2.2 GBq are used for remnant ablation in selected patients with low-risk DTCs. In these settings, rhTSH use should be preferred considering the better patient quality of life and the lower radiation exposure [39, 43–46].

The optimal RAI activity and stimulation method for adjuvant treatments in patients with intermediate-risk DTC are currently unknown [6]. Accordingly, the use of thyroid hormone withdrawal (THW) or rhTSH and administered RAI activity should be decided on an individual basis in a multidisciplinary setting. Our panel recommends rhTSH-stimulated administration of 2.2–3.7 GBq of RAI in intermediate-risk patients. Administration of ≥3.7 GBq of RAI is recommended in high-risk patients, preferentially after THW as, according to ATA 2015 guidelines, more controlled data from long-term outcome studies are needed before rhTSH preparation for RAI adjuvant treatment can be recommended for routine practice [3]. However, rhTSH stimulation is mandatory when physiologic TSH stimulation is precluded (e.g., pituitary diseases) or when THW is clinically contraindicated.

#### Key points and practical indications

Postoperative ablation/adjuvant RaIT administration (suggested RAI activities and stimulation protocols):

- Low-risk DTC (<2 cm): selective ablation RaIT (1.1–2.2 GBq) with rhTSH stimulation.
- Low-risk (>2 cm) and intermediate-risk DTC: adjuvant RaIT (2.2–3.7 GBq) with rhTSH stimulation.
- High-risk DTC: adjuvant RaIT (≥3.7 GBq) with THW stimulation.

## Long-term follow-up

Follow-up of DTC after primary treatment has evolved from standard to a more individualized approach that is currently based on ongoing risk assessment [3, 47]. Initial risk assessment is continuously modified and refined by the inclusion of assessments of response to therapy and disease course [3]. Serum Tg measurements [1, 48, 49], neck ultrasound [50, 51], and ^123/131^I-RAI Dx-WBS [51] are commonly used as primary tools for DTC follow-up.

Unstimulated and/or stimulated serum Tg measurement is currently the yardstick for monitoring patients with DTC after primary therapy. However, its usefulness is limited in patients who are positive for TgAb since serum Tg levels can be underestimated when measured using immunometric assays [52, 53].

Neck ultrasound offers several advantages and is a reliable method for detection of loco-regional persistent or recurrent DTC (i.e., thyroid bed and cervical lymph nodes), but is not without limitations [51]. Indeed, the number of neck ultrasounds providing false positive results, which increases the risk of unnecessary and expensive additional procedures, are far from negligible [54–56]. Accordingly, the use of neck ultrasound should be limited (particularly in low-risk DTC) and, in the absence of TgAb, reserved for patients with unstimulated serum Tg levels ≥1 ng/mL [55, 57].

According to ATA 2015 guidelines, Dx-WBS can be useful for patients with a high- or intermediate-risk of persistent disease, but should not be routinely used for the follow-up of other patients [3]. The use of Dx-WBS in low-risk patients is generally discouraged if serum Tg is undetectable and neck ultrasound is negative at the first response assessment. However, an analysis by Gonzalez-Carvalho et al. of a large series of patients followed for up to 25 years suggested that routine Dx-WBS is useful at the first follow-up (6–12 months after RaIT) in all cases [58]. Moreover, there is agreement on the relevant role of Dx-WBS in patients who are positive for TgAb [59], or with extra-thyroid uptake at post-therapy-WBS (pT-WBS) or large thyroid remnants precluding pT-WBS, and in selected cases based on individual risk profiles (e.g., isthmus location of malignant nodule). To date, the use of rhTSH and hybrid SPECT/CT has been shown to reduce patient discomfort and significantly improve the diagnostic performance of Dx-WBS using either ^131^I or ^123^I [60–62].

**Fig. 3:**
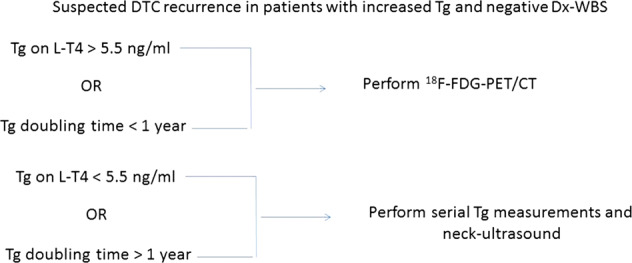
Assessment of patients with recurrent differentiated thyroid carcinoma (DTC) in relation to thyroglobulin (Tg) level [63]. ^18^FDG-PET/CT ^18^F-fluoro-2-deoxyglucose-positron emission tomography/computed tomography, Dx-WBS diagnostic whole-body scan, Tg-DT thyroglobulin doubling time

^18^F-FDG-PET/CT is the recommended first-line diagnostic procedure for anaplastic and other aggressive thyroid cancers, and is an important second-line procedure for DTC follow-up [3]. In the latter setting, ^18^F-FDG-PET/CT is especially recommended in patients with increasing Tg levels, negative ultrasound, and RAI imaging results [3]. Serum Tg thresholds used to select patients for PET/CT imaging vary widely in literature and should be optimized locally, taking into account patient characteristics and the serum Tg and TgAb assays used. Interestingly, evaluation of Tg doubling time can be used in addition to ^18^F-FDG-PET/CT (Fig. 3) [63]. Current data also suggest a role for ^18^F-FDG-PET/CT in patients with negative ultrasound and Dx-WBS who have increasing TgAb levels [64–66]. In addition, ^18^F-FDG-PET-CT is advised in patients with metastatic DTC, poorly DTC histotypes; to identify patients at highest risk for rapid progression and for evaluating the response to systemic and/or local treatments [1, 3].

### Key points and practical indications

- Follow-up after primary treatment is currently based on dynamic risk stratification, with neck ultrasound and measurement of Tg levels (either unstimulated or after TSH stimulation) being standard.
- Dx-WBS is especially suggested in patients with:
  - intermediate- or high-risk DTC;
  - extra-thyroid RAI uptake on pT-WBS;
  - large thyroid remnants reducing reliability of pT-WBS;
  - positive TgAb, limiting the validity of Tg as a DTC tumor marker.
- ^18^F-FDG-PET-CT should be considered for patients with negative post-treatment WBS or diagnostic WBS and neck ultrasound despite:
  - elevated basal or stimulated serum Tg levels (>5.5 ng/ml or >10 ng/ml, respectively) or serum Tg doubling time is <1 year (regardless of initial value);
  - increasing TgAb.
- In addition, 18F-FDG-PET-CT should be always considered:
  - in patients with poorly differentiated thyroid cancer;
  - in patients with metastatic disease to identify those at highest risk for rapid disease progression;
  - for evaluating the response to systemic and/or local treatments in patients with advanced disease.

## Metastatic DTC patients

Loco-regional and distant metastases can be detected at diagnosis, during postoperative assessment with ^123/131^I-RAI Dx-WBS, following RaIT, or during follow-up [3, 67]. Distant metastases are more frequent in patients with high-risk DTC [59]; however, they can also be found in patients with low-risk DTC [68, 69]. A summary of the risk factors associated with a worse prognosis in metastatic DTC is shown in Table 2 [70].

**Table 2:** Factors associated with worse prognosis in patients with metastatic differentiated thyroid carcinoma

Risk factors
Age >55 years
Male gender
Follicular histology
Distant metastases at diagnosis
Bone or combined distant metastases (e.g., bone and lung metastases)
RaIT-refractory disease

*RaIT* radioiodine therapy

Management of metastatic DTC should involve a multidisciplinary team and should be based both on local and systemic treatments [3, 71, 72]. In patients with RAI-avid metastatic disease, RaIT remains the treatment of choice [3], providing a favorable impact on overall survival and disease-free survival [73–76]. The administered RAI activity can be determined empirically or based on a dosimetric approach [72, 77, 78]. The empiric approach uses a fixed activity (i.e., 3.7–11.1 GBq), which is selected based on disease stage, age, burden of disease, and kidney function [61]. In addition, important limiting factors such as reduced bone marrow reserve (mainly in patients >70 years) and hampered lung function (especially in patients with disseminated lung metastases and in pediatric patients) should also be taken into account when choosing empiric activity [72]. This approach is simple, effective, and widely used in clinical practice. The potential limitations include over- or under-treatment and a hypothetical progressive loss of efficacy after repeated treatment [59, 72, 78–80].

The dosimetric approach uses patient-tailored activities according to the principles of As High As Safely Administrable [81] and/or As Low As Reasonably Achievable (ALARA) [34]. This approach is complex to implement, but offers a number of important theoretical advantages, including high rates of therapeutic success with a single treatment, the possibility of adjusting the radiation activity to the target volume, and increased safety for nontarget organs and tissues. Currently, due to a lack of consistent evidence, it is challenging to confirm a preference for the empiric or dosimetric strategy for RaIT [3]. However, the latter approach may be preferable in special conditions (e.g., renal failure, diffuse miliary lung metastases, and reduced bone marrow reserve) in order to reduce toxicity [82–85].

To date, regardless of the strategy used, THW is indicated in patients preparing for RaIT [3], with the use of rhTSH reserved for patients whose endogenous TSH stimulation is precluded (e.g., pituitary diseases) or clinically contraindicated [2, 4, 72, 86–88]. Note, if the dosimetric approach is chosen, RaIT must be performed in the same functional status (i.e., hypothyroidism or euthyroidism) that was used for dosimetric calculations.

In patients with RAI-avid metastatic DTC, RaIT should be repeated until a complete response is achieved (i.e., no abnormal RAI uptake at Dx- or pT-WBS and stimulated Tg levels below the functional sensitivity cut-off in absence of TgAb) or there is an adequate and durable response (i.e., slow or no progression of disease) as previously described [89, 90].

Conversely, RaIT should be stopped in patients with unresponsive, RaIT-refractory disease (Table 3) [72, 90]. In this light, some criteria were proposed in literature (e.g., negative RAI imaging, cumulative activity >22.2 GBq) [91, 92]. However, none of the proposed criteria is considered “sacrosanct” [90] and a comprehensive assessment that integrates all of the clinical, biochemical, and imaging data collected during patient management and follow-up should be done before stopping RaIT [90, 93].

**Table 3:** Definition and initial management of radioiodine therapy-refractory differentiated thyroid carcinoma

Recommendations
• A negative Dx-WBS or pT-WBS is not sufficient to classify a patient as RaIT-refractory.
• The quality of the various assessments performed should always be carefully checked.
• Patients with ≥1 negative lesion on Dx-WBS should not be considered refractory but should receive local treatment for WBS-negative lesions and RaIT for RAI-avid lesions.
• Assessment of structural response to treatment should not strictly adhere to the RECIST criteria but should be individualized by taking into account patient clinical status and wishes.
• The overall course of serum Tg levels should be evaluated, not absolute Tg levels.
• The duration of response to treatment should be recorded (<6 months or >12 months, or in between).
• The overall amount of ^131^I-RAI activity administered should be monitored.
• The frequency and severity of adverse events should be recorded.
• The cumulative administered ^131^I-RAI activity being above the suggested limit is not sufficient to define a patient as having RaIT-refractory disease.

*Dx-WBS* diagnostic WBS, *pT-WBS* post-therapy WBS, *RAI* radioiodine, *RaIT* radioiodine therapy, *RECIST* Response Evaluation Criteria In Solid Tumors, *Tg* thyroglobulin, *WBS* whole-body scan

### Key points and practical indications

- Management of metastatic DTC should involve a multidisciplinary team and should be based on both local therapy (e.g., surgical resection, radiofrequency ablation, cryoablation, and external beam radiation therapy) and systemic treatment (e.g., RaIT, levothyroxine suppressive therapy, targeted therapy, and cytotoxic chemotherapy).
- In patients with RAI-avid metastatic disease, RaIT may improve overall and disease-free survival and remains the treatment of choice.
- RAI activity to be administered can be determined empirically (3.7–11.1 GBq) or based on a dosimetric (individualized) approach; individualized dosimetry is preferable in selected cases (e.g., renal failure, diffuse miliariform lung metastases, reduced bone marrow reserve, and pediatric patients).
- THW is recommended to prepare patients with metastatic DTC for RaIT; however, rhTSH is mandatory when endogenous stimulation is precluded (i.e., pituitary diseases) or clinically contraindicated.
- The definition of RaIT-refractory patients (Table 3) is evolving toward an individualized and dynamic definition that can integrate all of the clinical, biochemical, and instrumental data collected during patient management and follow-up.

## Conclusions

The Martinique Principles should be implemented in routine clinical practice in order to optimize clinical management/outcomes in patients with DTC. Based on the clinical experience of five thyroid cancer experts, we provide a suggested approach for assessing and diagnosing thyroid nodules in clinical practice, as well as our recommendations for the use of surgery and postoperative RaIT, and long-term follow-up of patients with DTC. The multidisciplinary approach is particularly important in the management of patients with metastatic DTC, which should be individualized according to disease status and comorbidities. Comprehensive assessment of patients with suspected RaIT-refractory disease is recommended before stopping RaIT.
